# The Old and the New Vitalism

**Published:** 1896-03-21

**Authors:** 


### THE OLD AND THE NEW VITALISM.

Professor Burdon Sanderson, F.R.S., contributes to this month's *Science Progress* a very interesting article on "Ludwig and Modern Physiology," in which he describes the change from those old ideas of vitalism by which the processes of life used to be explained, and those chemico-physical conceptions of vital processes which in later years have held the field. The old vitalism of the first half of the century is easily explained. It was generally believed that, on the whole, things went on in the living body as they do outside of it, but when any difficulty arose in so explaining them the physiologist was ready at once to call in the aid of a "vital force." Such doctrines, however, rapidly gave way to the advance of knowledge as to the correlation of the physical sciences which took place in the forties. In 1847, Ludwig, on his first visit to Berlin, met three other men of his own age, viz., Helmholtz, du Bois-Reymond, and Brücke, who were destined to become his life-friends, and all of whom attained to the highest distinction. They were all full of the same enthusiasm. As Ludwig said when speaking of this visit, "We four imagined that we should constitute physiology on a chemico-physical foundation, and give it equal scientific rank with physics, but the task turned out to be much more difficult than we imagined."

It was then with such ideas that Ludwig entered on his work as a teacher and investigator, and it has been by his continued insistence on the sufficiency of ordinary laws to explain at any rate the processes of life, that his great influence on contemporary thought has been largely due.

The proof of the non-existence of a special "vital force" lies, as Professor Burdon Sanderson says, in the demonstration of the adequacy of the known sources of energy in the organism to account for the actual day by day expenditure of heat and work; in other words, on the possibility of setting forth an energy balance-sheet, in which the quantity of food which enters the body in a given period (hour or day) is balanced by an exactly corresponding amount of heat produced or external work done; and this has at length been successfully accomplished, and we have the experimental proof that in the process of life there is no production or disappearance of energy, and that food is the sole and adequate source of all we disengage, whether in the form of heat or of external work.

The old vitalism then is to be counted as dead, but under the term "Neovitalismus" a sort of reaction has recently attracted some attention. It is not now suggested that vital force makes the elements perform in opposition to ordinary laws, but it is maintained that under the influence of improved methods of research certain processes which had been regarded as entirely physical or chemical do not conform so precisely as they were expected to do to physical or chemical laws—that there is a something still unexplained. To the question, How is this? the "Neo-vitalists" answer at once that these abnormalities occur in consequence of cells acting in obedience not to physical laws but to vital ones—to internal laws which are special to themselves.

This, however, is but pushing the question one step further back, for before cell activities can be separated from chemico-physical processes it must be shown that they do not also, as does the body as a whole, use up energy in one form in the act of manifesting it in another. That matter in the form of living cells acts differently from the same matter in the form of dead ones everyone admits. Each cell is a special machine to do special work, which doubtless could not be done in any other way; but to say that, because we cannot as yet see the exact method by which this is done, and because its work is different from what can be done by anything else, its atoms are subject to different laws, cannot logically be separated from that older vitalism which fled in every difficulty to "vital force," was contented with explaining one unknown thing by another and is now discredited.

